# Not All Electrode Channels Are Needed: Knowledge Transfer From Only Stimulated Brain Regions for EEG Emotion Recognition

**DOI:** 10.3389/fnins.2022.865201

**Published:** 2022-05-24

**Authors:** Hayford Perry Fordson, Xiaofen Xing, Kailing Guo, Xiangmin Xu

**Affiliations:** ^1^School of Electronic and Information Engineering, South China University of Technology, Guangzhou, China; ^2^School of Future Technology, South China University of Technology, Guangzhou, China

**Keywords:** emotion recognition, transfer learning, brain region, channel selection, EEG, domain adaptation

## Abstract

Emotion recognition from affective brain-computer interfaces (aBCI) has garnered a lot of attention in human-computer interactions. Electroencephalographic (EEG) signals collected and stored in one database have been mostly used due to their ability to detect brain activities in real time and their reliability. Nevertheless, large EEG individual differences occur amongst subjects making it impossible for models to share information across. New labeled data is collected and trained separately for new subjects which costs a lot of time. Also, during EEG data collection across databases, different stimulation is introduced to subjects. Audio-visual stimulation (AVS) is commonly used in studying the emotional responses of subjects. In this article, we propose a brain region aware domain adaptation (BRADA) algorithm to treat features from auditory and visual brain regions differently, which effectively tackle subject-to-subject variations and mitigate distribution mismatch across databases. BRADA is a new framework that works with the existing transfer learning method. We apply BRADA to both cross-subject and cross-database settings. The experimental results indicate that our proposed transfer learning method can improve valence-arousal emotion recognition tasks.

## 1. Introduction

**Figure 1:**
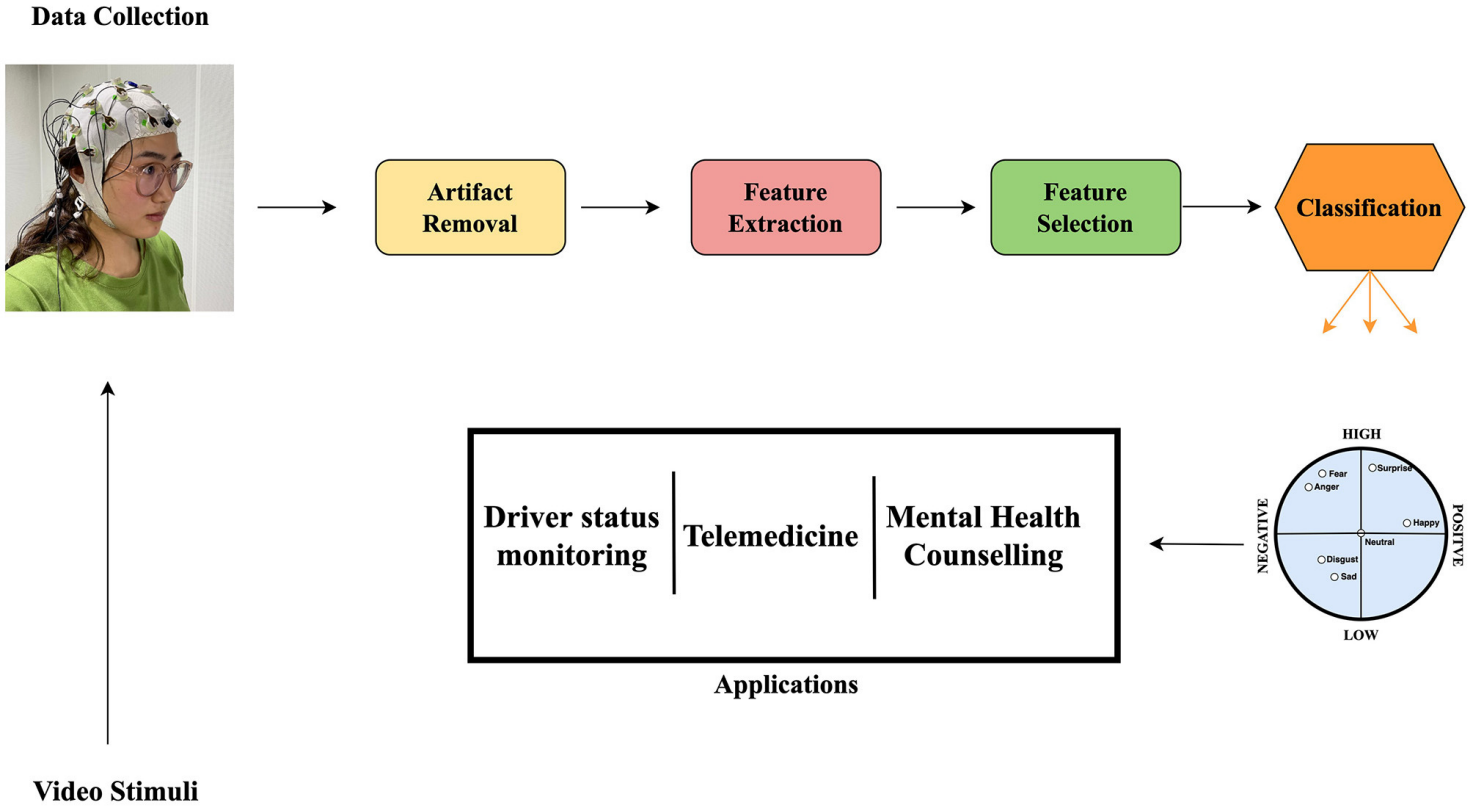
Illustration of an affective brain-computer interfaces (aBCI) and its applications (Fordson et al., 2021). Source for the photo top left: Center for Human Body Data Science, School of Electronic and Information Engineering, South China University of Technology.

Emotions play an important role in Human-Human Interaction by creating pathways for individuals to learn and adapt to behaviors (Ko et al., 2021). Human-computer interaction should be designed in such a way that better interacts with users, behavior and emotions and respond accordingly (Principi et al., 2021; Yun et al., 2021). Emotion recognition from Electroencephalograph (EEG) signals among many other physiological methods shown to be more advantageous in its non-invasiveness and reliability. EEG is a widely used medical instrument for recording electrical currents generated by brain activity (Kwak et al., 2021). An affective brain-computer interface (aBCI) presents stimuli of different kinds to subjects by taking neural signals in a form of EEG recordings. EEG-based aBCI emotion recognition has gained research attention recently because of its rapidly growing field with multiple interdisciplinary applications (Alarcão and Fonseca, 2019; Torres et al., 2020). Figure 1 is an illustration of an aBCI and some of its applications. However, EEG signals have high inter-subject variations and this creates problems in designing models that generalize well across subjects. Conventionally, data are collected individually for each subject and a classifier is trained specifically for them rather than formulating a model with a classifier that can generalize well on all subjects concurrently. Another generalization problem arising from aBCI is the same subject session-to-session variation (Zheng and Lu, 2015). Domain adaptation (DA) methods can solve this issue (Yan et al., 2018). In order to establish an EEG database that fully elicits human emotions and meets a required classification task, some studies collect data for a single subject in multiple trials or sessions. Data collected per session can be reckoned as somewhat a new task because of the non-stationary characteristics of EEG signals (Shen and Lin, 2019). These require a great deal of time-consuming and re-calibration processes (Fdez et al., 2021) to minimize the variations in the subject-to-subject data and the session-to-session data for a single subject. Over the years, studies have focused on publicly available affective databases to assist researchers in designing and modeling their own affective state estimation methods (Koelstra et al., 2012; Soleymani et al., 2012; Katsigiannis and Ramzan, 2018; Subramanian et al., 2018; Zheng et al., 2019). These databases are collected using different stimulants, equipment, experimental environments and protocols, and target labels among many other different technical discrepancies. These differences are unique to a particular database but create research challenges when designing and building models intended to adapt across databases.

Transfer learning (TL) focuses on leveraging and storing knowledge acquired from a source domain task and applying it to a target domain task that may be different but of a related problem without the need to learn from scratch (Pan et al., 2011; Niu et al., 2021). TL compensates for inter-subject variability evidenced in EEG feature dispersion as a covariate shift for aBCI to increase confidence in classification performance in comparison to non-transfer learning tasks (Saha and Baumert, 2020). The study (Lin, 2020) proposes a TL model, robust principal component analysis (RPCA) to Single Day (sD) and Multiple Day (mD) data dealing with inadequate labeled data and concurrently solving inter and intra-individual differences. Pan et al. (2011) proposed a transfer component analysis (TCA), for domain adaptation that finds representation from feature nodes. The study by Yan et al. (2018) proposed a maximum independence domain adaptation (MIDA) technique to tackle different distributions of training and test data. Fernando et al. (2013) introduced a subspace alignment (SA) method mapping function which aligns source subspaces to target ones described by eigenvectors. Unlike image processing (Su, 2021), EEG-based emotion recognition requires a lot of time, effort, and equipment to collect data. Therefore, in order to reduce the constraint on aBCI systems, TL is of great importance. It has been widely used in EEG-based emotion recognition and makes good progress in dealing with the cross-domain scenarios problems of EEG signal (Li et al., 2021). However, to the best of our knowledge, all existing transfer learning methods for EEG treats all channels the same while actually different EEG channel plays a different role. It is more reasonable to transfer knowledge differentially according to channel locations.

Channel selection that uses only part of the channels is a widely used pre-processing step in EEG signal analysis (Alotaiby et al., 2015; Boonyakitanont et al., 2020). It can reduce overfitting which is due to the utilization of unnecessary channels. Various channel selection methods for EEG-based emotion recognition have been proposed, such as measuring the contribution of each channel (Yan et al., 2020), selecting according to classification performance (Özerdem and Polat, 2017), excluding channels least correlated with the emotional state (Dura and Wosiak, 2021), normalized mutual information (Wang, 2019), and weight distribution of trained neural network (Zheng and Lu, 2015). They all select channels by analyzing EEG data or model parameters. EEG signal for emotion recognition is usually stimulated by visual or auditory materials. However, the prior knowledge of brain regions for visual and auditory stimuli is overlooked in channel selection.

In this study, we transfer knowledge from only stimulated brain regions according to prior neuroanatomy knowledge (Sotgiu et al., 2020) for EEG emotion recognition. Our approach does not only improve the accuracy of emotion recognition but provides new insight into EEG transfer learning.

Also, we show that for EEG signals, transferring knowledge in a finer way instead of treating all the channels the same will improve the performance. We reveal the effectiveness of applying prior knowledge of EEG signals for channel selection, unlike previous studies that utilized all EEG signals from channels that may not be needed. Our proposed improved transfer learning method not only works for cross-subject scenarios but also for cross-database scenarios.

## 2. Materials and Methods

### 2.1. Related Work

In this section, we review studies relating to aBCI using EEG signals and transfer learning methods that are most related to our proposed study. We presented studies on cross-subject, cross-database, and EEG channel selection for emotion classification. Moreover, we remonstrate the uniqueness of our article to distinguish it from recently published studies.

#### 2.1.1. Transfer Learning for EEG-Based Emotion Recognition

##### 2.1.1.1. Cross-Subject

The amount of available training data in an aBCI affects models' performance. However, the statistical distribution of training data varies across subjects as well as across trials/sessions within subjects, thus limiting the transferability of the training model between them (Lin, 2020). Azab et al. (2019) proposed a novel transfer learning system that reduces calibration time yet maintains classification accuracy by incorporating previously recorded data from other subjects when only few subject-specific sessions are available for training. Standard proposed methods dealing with subjects' differences are mostly based on common spatial pattern (CSP) (Martin-Clemente et al., 2019) which is a dimensionality reduction technique that linearly projects training data onto directions maximizing or minimizing the variations between them. CSP filtering methods reveal more information about the data and result in high efficiency values. Wu et al. (2020) proposed a TL protocol for closed-loop BCI systems and suggests data alignment before spatial filtering to make data from different subjects consistent and facilitate succeeding TL algorithms. Li et al. (2020) identified the problem of time consumption and build models for new subjects to reduce the demand for labeled data. Their method includes source selection and mapping destinations. They used style transfer mapping (STM) to reduce EEG differences between source and target data and explore mapping destination settings. The studies of He and Wu (2020) proposed an approach to align EEG trials from several subjects in Euclidean space by reducing variations that improve the learning performance of new incoming subjects. Their method aligns EEG sessions well into Euclidean space, employs low computational cost, and exhibits the usefulness of unsupervised classification (Rouast et al., 2021).

##### 2.1.1.2. Cross-Database

Cross-database involves using two or more databases in building an effective aBCI for emotion recognition. We have searched numerous academic databases in an attempt to find works in this regard in addition to using transfer learning methods. However, difficult the search was, we have found some related works. Jayaram et al. (2016) introduced a model for transfer learning in EEG-based BCI that exploits multiple subjects and/or sessions shared structures between training data to increase performance. They demonstrated their method's usefulness in limiting time consumption and its capability of outperforming comparable methods on identical datasets. Rodrigues et al. (2019) present a transfer learning approach that deals with statistical variations of EEG signals collected from different subjects in different sessions. Their article proposed a Procrustes analysis method to match the statistical distributions of two datasets (simulated data and real data) using simple geometrical transformations over data points. Cimtay and Ekmekcioglu (2020) investigated pre-trained neural network models trained on the SEED dataset (Zheng and Lu, 2015) and tested them on the DEAP dataset (Koelstra et al., 2012) that yields a reasonable mean prediction accuracy. The study of Lan et al. (2019) focused on comparative studies on SEED and DEAP datasets. They used existing domain adaptation (DA) techniques on these datasets and reported their effectiveness in an unsupervised setting (Fernando et al., 2013).

##### 2.1.1.3. EEG Channel Selection

The study by Daoud and Bayoumi (2019) uses channel selection methods to identify relevant EEG channels using a semi-supervised approach based on transfer learning. In order to simplify the training model, the authors of Ramadhani et al. (2021) used integrated selection (IS) to remove irrelevant EEG channel signals which further improved the performance of an aBCI system. The article by Basar et al. (2020) used welch power spectral density-based analysis to see the effects of CSP algorithms on EEG band and channel relationship, its neural efficacy, and emotional stimuli types. Also, Cao et al. (2020) selected EEG channels according to Fisher criteria and trained their model on a convolutional neural network based on parameter transfer. The selected channel features in comparison with non-channel selection demonstrate a higher accuracy performance.

The works of transfer learning and channel selection on cross-database lack sufficient investigation even though they relax research restraints of a typical aBCI. Previous studies on EEG-based transfer learning do not investigate two real EEG databases with significantly related components. They also do not focus on investigating selecting channels that contribute most to improving affective computing systems. They either use simulated data vs. real data (Yan et al., 2018; Rodrigues et al., 2019) or try to reduce components of one database to the other (Lan et al., 2019). Also, they do not focus on stimulated brain regions and select channels that can contribute meaningful insight. Instead, they extract features from all channels and follow traditional feature combination techniques in recognition tasks. Our article utilizes two databases with a significant focus on selecting stimulated brain regions that can affect participant responses in emotion elicitation. Emotional responses to videos in both databases are correlated with both employing dimensional models of valence and arousal. The motivation of this article is to adapt feature-space transfer learning and parameter-space transfer learning to new subjects in one database by decreasing variations within subject-to-subject and new databases by also decreasing database-to-database variations. This will in turn produce a robust classification method for affective BCI.

### 2.2. Database

In this article, we utilized two publicly available databases, MAHNOB-HCI (Soleymani et al., 2012) and DEAP (Koelstra et al., 2012). We chose these databases because of the differences and the similarities they share. For example, similarities,—they are both collected with the same type of device and, differences,—the MAHNOB-HCI contains both audio and video while the DEAP only contains audio. As we mentioned in Section 1, transfer learning involves creating new models by fine tuning previously trained models and adapting knowledge learned while solving one problem and applying it to a different but related problem. Therefore, these two databases are different but related. Furthermore, our study is interested in the robustness of an applicable aBCI in a cross-database fashion. We plan to train our model on one database with different subjects and test it on another database with new subjects. We also intend to investigate the possible effects of transfer learning techniques on the prospect of heightening classification accuracy.

The MAHNOB-HCI database is recorded in response to affective stimuli with the common goal of recognizing emotions and implicit tagging. It consists of 30 subjects (13 men, 17 women). They were aged between 19 and 40 years (mean age of 26.06). Unfortunately, three subjects' data were lost due to technical errors, thus, 27 subjects (11 men, 16 women) data were considered for processing. The subjects watched 20 emotional movie videos and self reported felt emotions using arousal, valence, dominance, and predictability in addition to emotional keywords. The database comprises 32-channel EEG signals in accordance with the international 10-20 system. The EEG signals were recorded using the Biosemi Active II system with active electrodes at a 1,024 Hz sampling rate and downsampled to 256 Hz to reduce memory and processing cost.

The DEAP database is recorded for the analysis of human affective states. A total of 32 subjects participated in this experiment (16 men, 16 women). They are aged between 19 and 37 years (mean age of 26.9). The subjects watched as stimuli 40 1-min long excerpts of music video while their physiological signals are being collected. After each trial, participants rated each music video in terms of their level of arousal, valence, dominance, liking, and familiarity. The rating values comprise a continuous scale of 1–9 for arousal, valence, dominance, and liking, and a discrete scale of 1–5 for familiarity. The EEG signals were recorded with 32-channel electrodes placed according to the international 10-20 system at a sampling rate of 512 Hz and downsampled to 128 Hz. Table 1 sums up the important technical specification of the two EEG databases.

**Table 1:** Specification comparison and database content summary of MAHNOB-HCI and DEAP.

**Item content**	**MAHNOB-HCI (Soleymani et al., 2012)**	**DEAP (Koelstra et al., 2012)**
No. of subjects	27 (11 males and 16 females)	32 (16 males and 16 females)
Stimuli effect	Audio and visual	Audio and visual
Stimuli selection method	Movie clip	Music video
EEG Device	Biosemi Active II	Biosemi Active II
No. of channels	32	32
Sampling rate	Collected at 1,024 Hz, downsampled to 256 Hz	Collected at 512 Hz, downsampled to 128 Hz
Rating scales	Arousal and valence	Arousal and valence
Rating values	Discrete scale of 1–9	Discrete scale of 1–9
No. of data collection sessions for one subject	1	1
No. of videos	20	40
Video length	Between 34.9 and 117 s	63 s

### 2.3. Methodology

**Figure 2:**
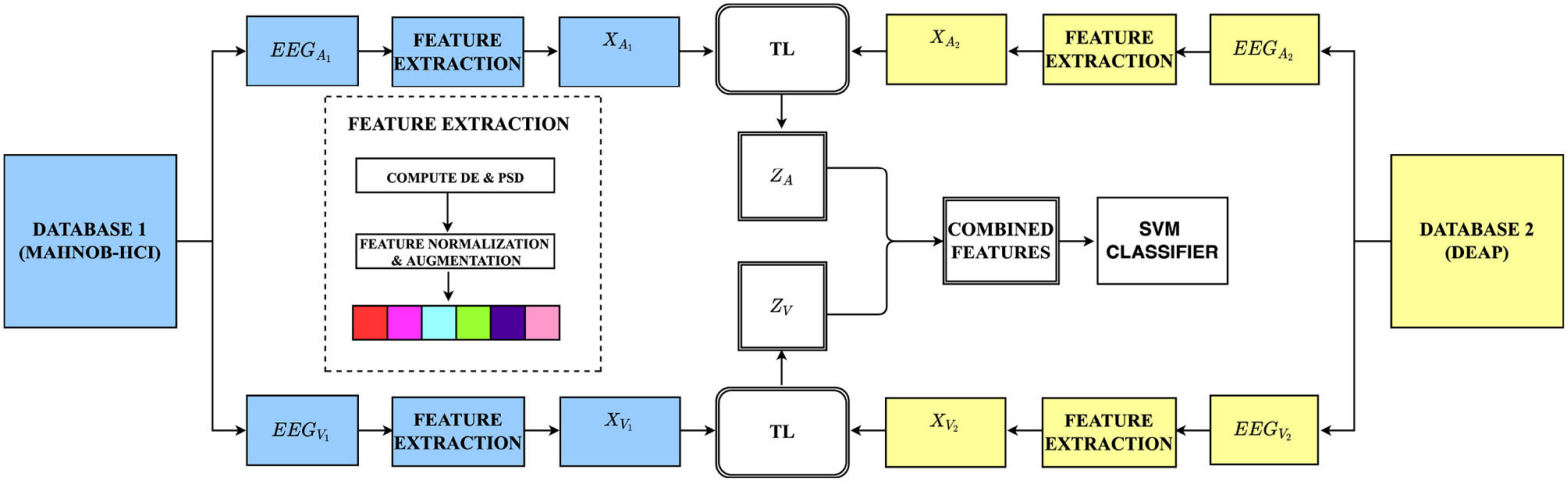
The overall framework of our method: We extracted and selected features according to auditory and visual channels on both databases. Using transfer learning (TL) techniques, we transferred knowledge separately from the MAHNOB-HCI database and employed a feature concatenation method in an attempt to boost performance accuracy in the DEAP dataset (*EEG*_*A,V*_, EEG auditory or visual data; *X*_*A,V*_, auditory or visual input features; *Z*_*A,V*_, combined auditory or visual features).

This section introduces our unsupervised transductive learning approach to this study. We present in the sub-sections how data are formulated, which features are extracted, and our proposed brain region aware domain adaptation (BRADA) method. Taking transfer learning between two databases as examples, the illustration of BRADA is shown in Figure 2.

#### 2.3.1. Visual and Auditory Channels

**Figure 3:**
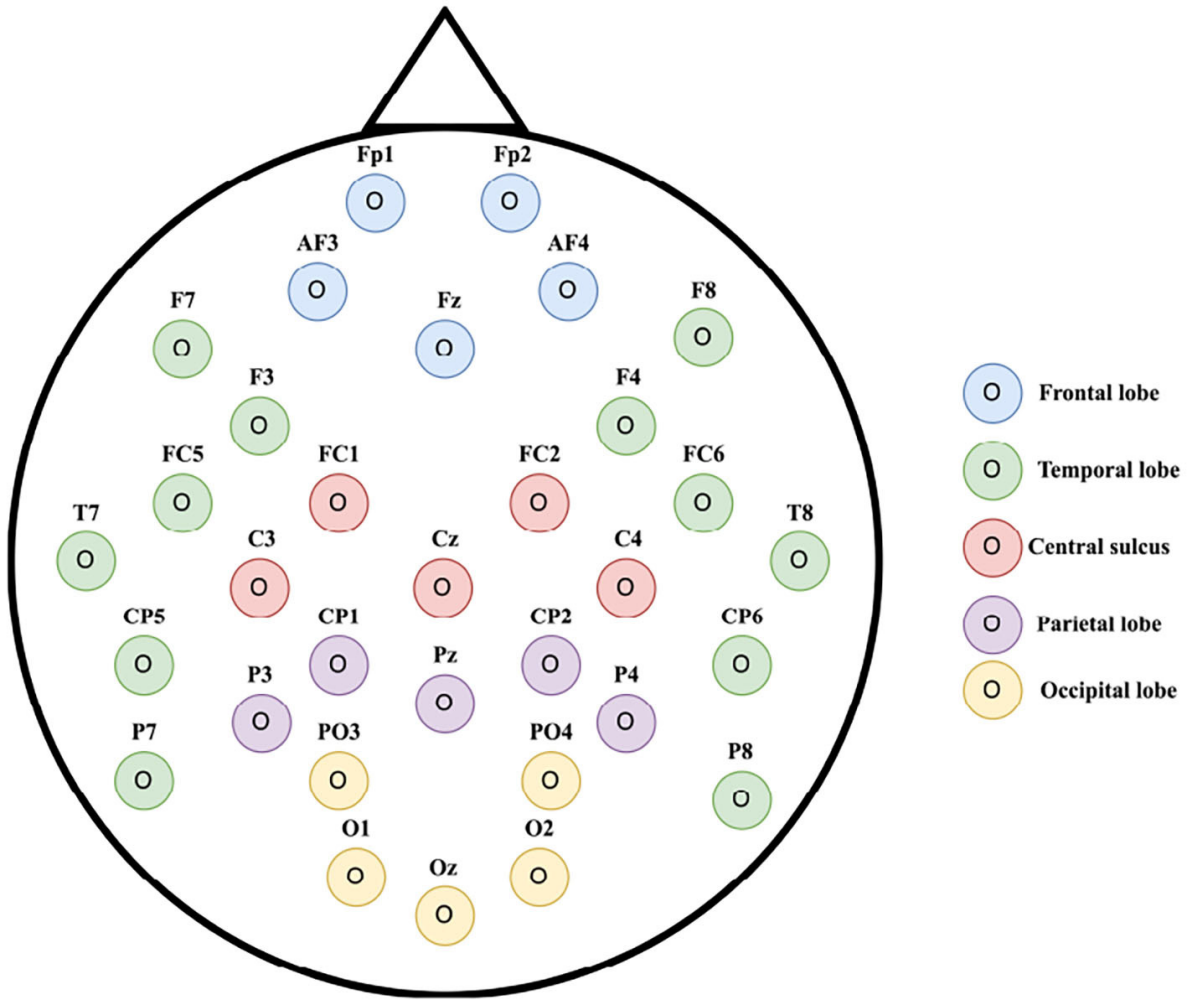
Feature selection on electrode placement of 32 channel Electroencephalographic (EEG) according to the international 10-20 system (best seen in color) (Alarcão and Fonseca, 2019).

According to neuroanatomy (Sotgiu et al., 2020), we divide the international 10-20 EEG system into five regions (the frontal, parietal, occipital, temporal lobes, and the central sulcus). Figure 3 gives an illustration. Among them, the occipital lobe's primary function is to control vision and visual processing and the temporal lobe is related to the perception and recognition of auditory stimuli, speech, and memory. We call electrodes located in the occipital lobe (*PO*3, *PO*4, *O*1, *Oz*, and *O*2) and electrodes located in the temporal lobe (*F*7, *F*8, *F*3, *F*4, *FC*5, *FC*6, *T*7, *T*8, *CP*5, *CP*6, *P*7, and *P*8) as visual and auditory channels. We only extract features for these channels and transfer knowledge for the specific region separately.

#### 2.3.2. Feature Extraction

In this article, we adopt Differential Entropy (DE) (Lan et al., 2019) and Power Spectral Density (PSD) (Fang et al., 2021; Zhu and Zhong, 2021) features for emotion classification. These features have been extensively used in EEG-Based emotion recognition (Zheng et al., 2015; Arnau-Gonzalez et al., 2021; Zhu and Zhong, 2021). The feature includes DE and PSD from theta (4 Hz < *f* <8 Hz), slow alpha (8 Hz < *f* <10 Hz), alpha (8 Hz < *f* <12 Hz), beta (12 Hz < *f* <30 Hz), and gamma (30 Hz < *f*) bands of EEG signal. Therefore, for 32 electrode baseline classification, the dimension *m* of the feature vector is 32 × 5+32 × 5 = 320 features. Specifically, for auditory channel selection, the dimension *m*_*A*_ of the feature vector is 12 × 5 + 12 × 5 = 120; for visual channel selection, the dimension *m*_*V*_ of the feature vector is 5 × 5 + 5 × 5 = 50 features. After feature extraction, we use min-max normalization to scale the feature value to a proper range as follows:


(1) $$x^{\prime }=\frac{x-\min(x)}{\max(x)-\min(x)}$$

where **x** is the original feature vector, and **x** ′ is the normalized feature value.

#### 2.3.3. Brain Region Aware Domain Adaptation

Assume $X_{s}\in {\mathbb{R}}^{m\times n_{s}}$ and $X_{t}\in {\mathbb{R}}^{m\times n_{t}}$ are respectively the normalized source and target domain data, where *m* is the feature dimension, and *n*_*s*_ and *n*_*t*_ are the sample numbers of source and target domains. Usually, data from different domains follow different distributions. Domain adaptation (Pan and Yang, 2010) is to find a domain-invariant subspace such that a classifier trained on the source domain can be directly applied to the target domain. That is to say, we want to reduce the discrepancies between the source and target domains by transforming $X=\left[X_{s},X_{t}\right]\in {\mathbb{R}}^{m\times n}$ to $Z=\left[Z_{s},Z_{t}\right]\in {\mathbb{R}}^{h\times n}$, where *h* is the dimension of the domain-invariant subspace, and *n* = *n*_*s*_+*n*_*t*_ is the total number of samples.

We utilize maximum independence domain adaptation (MIDA) (Yan et al., 2018) for feature transformation of both brain regions. Following Yan et al. (2018) and Lan et al. (2019), we define domain features to describe the background information of a sample and maximize the independence between the projected features and the domain features. The domain feature $d\in {\mathbb{R}}^{m_{d}}$ of a sample is defined by a one-hot encoding vector with *d*_*i*_ = 1 if the sample is from subject *i* and 0, otherwise. Following Lan et al. (2019), we encode the background information with feature augmentation by concatenation. The intention of the augmentation operation is to learn information-specific subspaces and to deal with time varying drift. The main difference between our method and Lan et al. (2019) is that we augment features of audio and video channels separately. Denote audio channel features and video channel features after normalization as *X*_*A*_ and *X*_*V*_, respectively. Then, the augmented features are represented by


(2) $$\begin{array}{r} {\bar{X}}_{A}=\left[\begin{array}{c} X_{A}\\ D \end{array}\right]\in {\mathbb{R}}^{\left(m_{A}+m_{d}\right)\times n} \end{array}$$

and


(3) $$\begin{array}{r} {\bar{X}}_{V}=\left[\begin{array}{c} X_{V}\\ D \end{array}\right]\in {\mathbb{R}}^{\left(m_{V}+m_{d}\right)\times n}. \end{array}$$

For simplification, we use *A*/*V* to denote either *A* or *V*. We use the kernel trick to project ${\bar{X}}_{A/V}$ to the desired subspace. Denote $\phi \left({\bar{X}}_{A/V}\right)$ as the mapping function. The transformed features are represented by a linear combination of the mapped features, i.e.,


(4) $$\begin{array}{r} Z_{A/V}={\tilde{W}}_{A/V}^{T}\phi \left({\bar{X}}_{A/V}\right). \end{array}$$

Following kernel dimension reduction methods (Kempfert et al., 2020), ${\tilde{W}}_{A/V}^{T}$ is constructed by a linear combination of all samples in $\phi \left({\bar{X}}_{A/V}\right)$. Then, we have


(5) $$\begin{array}{r} Z_{A/V}={\left(\phi \left({\bar{X}}_{A/V}\right)W_{A/V}\right)}^{T}\phi \left({\bar{X}}_{A/V}\right)=W_{A/V}^{⊤}K_{{\bar{X}}_{A/V}}, \end{array}$$

where *W*_*A*/*V*_ is the linear transformation matrix to be determined and $K_{{\bar{X}}_{A/V}}=\phi \left({\bar{X}}_{A/V}\right)\phi \left({\bar{X}}_{A/V}^{T}\right)$ is the kernel matrix of $\phi \left({\bar{X}}_{A/V}\right)$. By kernel trick, we can compute $K_{{\bar{X}}_{A/V}}$ with kernel function and does not need the explicit ϕ function. In this article, we use the polynomial kernel function.

As in Lan et al. (2019), we use the Hilbert-Schmidt independence criterion (HSIC) (Gretton et al., 2005) to measure the dependence between *Z*_*A*/*V*_ and *D*, which can be conveniently estimated by


(6) $$\begin{array}{r} HSIC\left(Z_{A/V},D\right)={\left(n-1\right)}^{-2}tr\left(K_{Z_{A/V}}HK_{D}H\right), \end{array}$$

where $K_{Z_{A/V}}={\left(W_{A/V}^{⊤}K_{{\bar{X}}_{A/V}}\right)}^{T}W_{A/V}^{⊤}K_{{\bar{X}}_{A/V}}$ and $K_{D}=D^{T}D$ are kernel matrices of *Z*_*A*/*V*_ and *D*, $H=1-n^{-1}1_{n}1_{n}^{⊤}\in {\mathbb{R}}^{n\times n}$ is the centering matrix, and **1**_*n*_ is an *n*-dimensional vector full of ones.

Following Yan et al. (2018) and Lan et al. (2019), we simultaneously maximize the variance of the projected data and their independence from the domain features. By omitting the scaling factor, the final objective function is given by:


(7) $$\begin{array}{r} \begin{array}{c} \max\;-tr\left(\overset{⊤}{\underset{A/V}{W}}K_{{\bar{X}}_{A/V}}HK_{D}HK_{{\bar{X}}_{A/V}}W_{A/V}\right)\\ +\mu \;tr\left(W_{A/V}^{⊤}K_{{\bar{X}}_{A/V}}HK_{{\bar{X}}_{A/V}}W_{A/V}\right)\\ s.t.\;W_{A/V}^{⊤}W_{A/V}=I \end{array} \end{array}$$

where μ>0 is a trade-off parameter. Here, we add an orthogonal constraint to the projection matrix *W* to ease the optimization. The solution to (7) can be obtained in a closed form by finding the *h* eigenvectors of $K_{{\bar{X}}_{A/V}}\left(-HK_{D}H+\mu H\right)K_{{\bar{X}}_{A/V}}$ corresponding to the top-*h* eigenvalues.

After obtaining *W*_*A*/*V*_, we compute the features of audio and visual channels by Equation (5). The final feature *Z* is then composed of the concatenation of *Z*_*A*_ and *Z*_*V*_, i.e., $Z={\left[Z_{A}^{T},Z_{V}^{T}\right]}^{T}$. The algorithm of our proposed brain region aware domain adaptation is summarized in Algorithm 1. With the extracted features and their given labels, we apply a support vector machine (SVM) (Burges, 1998) for classification.

**Table:** Algorithm 1 BRADA

**Input:** Feature matrix of all samples *X*; ϕ(·); hyper-parameters *h* and μ.
**Output:** Projected samples *Z*.
**Procedure:**
1: Avail domain features according to information-specific space from both databases.
2: Utilize MIDA to transform features of both brain regions, thus, auditory and visual channels.
3: Extract features separately into auditory and visual components as *X*_*A*_ and *X*_*V*_.
4: Combine them to obtain *X* features.
5: Normalize the features to obtain *X*′.
6: Augment the normalized features with domain features as in (2) and (3) to obtain $\bar{X}$.
7: Compute kernel matrices *K*_*Z*_*A*/*V*__ and *K*_*D*_ according to (6).
8: Optimize (7) to get *W*, namely the eigenvectors of $K_{{\bar{X}}_{A/V}}\left(-HK_{D}H+\mu H\right)K_{{\bar{X}}_{A/V}}$ in accordance to the largest *h* eigenvalues.
9: $Z={\left[Z_{A}^{T},Z_{V}^{T}\right]}^{T}$.

## 3. Results

This section demonstrates the effectiveness of BRADA in cross-subject and cross-database settings. We first show that BRADA is compatible with classical transfer learning methods, TCA (Pan et al., 2011), SA (Fernando et al., 2013), and MIDA (Yan et al., 2018) in both cross-subject and cross-database settings for EEG emotion recognition, and then we conduct ablation experiment to show the effectiveness of both brain regions, feature normalization and augmentation, and kernel function.

Baseline denotes applying SVM to the extracted features of all the channels directly. TCA, SA, and MIDA denote applying these methods to the extracted features of all the channels and then classifying the transformed features by SVM. We also replace MIDA in BRADA with the other transfer learning methods, denoted by “BRADA-” followed by a method name. Note that we use a polynomial kernel in Algorithm 1, but the original method (Yan et al., 2018) uses a linear kernel. Without confusion, we use BRADA to denote Algorithm 1 and use BRADA-MIDA to denote using a linear kernel. Without specification, all the transfer learning methods adopt a linear kernel. For all the methods, we set the trade-off parameter μ to 1, and the latent subspace dimension *h* is searched through {10, 20, ..., 100}.

For MAHNOB-HCI, since data of three subjects could not be validated, data from 27 subjects who had sufficient completed trials were used. Physiological responses recorded with EEG of five hundred and forty samples were collected over the database, 27 × 20 = 540 samples. In DEAP, data from all 32 subjects were used. Physiological responses recorded with EEG of one thousand two hundred and eighty samples were collected over the database, 32 × 40 = 1, 280 samples.

### 3.1. Cross-Subject Transfer Learning

For the cross-subject setting, we apply leave-one-out cross-validation on each database, i.e., one subject is chosen as the test set, and the remaining subjects are used for training. Each subject constitutes a single domain, and thus, our approach is formulated in a multi-source domain setting. Since our method is unsupervised, it is not constrained by a domain number. Thus, it can also operate in a single source domain multi-target domain mode.

The classification accuracy of both databases is reported in Table 2. We can see that BRADA achieves the best performance. Compared with baseline, all transfer learning methods with all the channels improve the results for MAHNOB-HCI. However, for DEAP, the transfer learning methods result in a negative transfer, i.e., result degradation. This is evident in the study of Chai et al. (2017) which mentions negative transfer in hindering domain adaptation methods from the successful operation of DEAP. Nevertheless, taking these methods into the framework of BRADA, the results are consistently improved. BRADA can effectively improve classification accuracy by 10.4–22.8%, suggesting that individual differences in all subjects are significantly reduced. Statistical significance analysis performed shows that accuracy improvements are significant (*t*-test, *p* < 0.05). This strongly suggests that the brain regions of different functions should be separately treated and not all channels are needed for EEG emotion recognition. We should emphasize that performance gain is significant compared to all the other methods.

**Table 2:** Cross-subject classification accuracy (%) on MAHNOB-HCI and DEAP.

**Method**	**MAHNOB-HCI**		**DEAP**	
	**Arousal**	**Valence**	**Arousal**	**Valence**
Baseline	57.2	55.5	57.5	62.1
TCA (Pan et al., 2011)	57.6	58.3	47.8	46.3
SA (Fernando et al., 2013)	57.3	56.4	42.0	42.9
MIDA (Yan et al., 2018)	58.5	62.1	48.6	48.2
BRADA-TCA	68.3	69.1	63.1	65.7
BRADA-SA	64.6	65.2	63.0	65.6
BRADA-MIDA	69.9	69.6	63.5	69.2
BRADA	**75.5**	**78.3**	**71.9**	**72.5**

*The bold values indicated the highest accuracy obtained*.

### 3.2. Cross-Database Transfer Learning

The previous sub-section presents the use of domain adaptation techniques which reduces inter-subject variations and improves classification performance on a single database. Here, we further present our study in a cross-database setting, i.e., the latent subspace is learned from all samples of both databases, and training and testing are applied to different databases. Note that this is a harder task because the differences between the samples are not only from personality and experimental sessions but also from equipment and experimental protocol. Each subject constitutes a single domain and thus this setting is multi-source and multi-target. For simplicity, we refer to MAHNOB-HCI as A and DEAP as B. We report the results of training on A and testing on B, and the reverse order in Table 3. We can see that, under both training settings, BRADA and its variants outperform their counterparts that take all the channels as input. Compared to the baseline accuracies with no domain adaptation method, BRADA can effectively improve classification accuracy by 22.6–33.1%, suggesting that individual differences in all databases are significantly reduced. Statistical significance analysis performed shows that accuracy improvements are significant (*t*-test, *p* < 0.05). This again verifies that selecting channels according to the stimuli sources can effectively reduce noise in EEG signals and transfer learning should be conducted separately for different brain regions.

**Table 3:** Cross-database classification accuracy (%) on MAHNOB-HCI and DEAP.

**Method**	**A → B**		**B → A**	
	**Arousal**	**Valence**	**Arousal**	**Valence**
Baseline	51.2	49.3	44.7	45.4
TCA (Pan et al., 2011)	49.5	46.5	58.5	58.6
SA (Fernando et al., 2013)	41.5	40.5	48.4	53.7
MIDA (Yan et al., 2018)	52.7	55.6	49.4	50.1
BRADA-TCA	64.0	68.4	69.6	70.5
BRADA-SA	63.1	68.2	65.7	66.2
BRADA-MIDA	63.7	69.2	70.8	71.6
BRADA	**73.8**	**75.9**	**76.2**	**78.5**

*The bold values indicated the highest accuracy obtained*.

### 3.3. Ablation Study

In order to effectively evaluate the BRADA algorithm and audio-visual feature combination for classification, we perform an ablation study to better understand our contributions and compare the effects of channel selection, normalization/augmentation, and kernel function selection on baseline features and existing models.

#### 3.3.1. Effect of Channel Selection

The baseline model consists of features from 32 electrode channels—including channels that may not be needed. In our proposed methodology, we selected features from auditory and visual brain regions where we believe participants are more stimulated. The results are reported in Tables 4, 5. Combining both brain regions with BRADA performs better than transferring knowledge from single brain regions or through direct concatenation.

**Table 4:** Cross-subject classification accuracy (%) comparison with single brain region on MAHNOB-HCI and DEAP databases.

**Methods**	**Auditory channels**				**Visual channels**				**Auditory + Visual channels**			
	**MAHNOB-HCI**		**DEAP**		**MAHNOB-HCI**		**DEAP**		**MAHNOB-HCI**		**DEAP**	
	**Arousal**	**Valence**	**Arousal**	**Valence**	**Arousal**	**Valence**	**Arousal**	**Valence**	**Arousal**	**Valence**	**Arousal**	**Valence**
TCA (Pan et al., 2011)	59.7	54.4	54.6	54.9	56.4	55.8	49.8	53.3	60.3	59.9	58.7	55.6
SA (Fernando et al., 2013)	46.5	46.2	43.2	46.7	42.7	45.6	41.1	43.7	48.2	45.9	47.2	46.9
MIDA (Yan et al., 2018)	60.3	59.1	58.3	62.3	59.6	55.7	52.9	51.0	62.3	64.0	61.5	60.2
MIDA-POLY	56.2	57.9	57.4	60.5	55.8	54.6	48.9	48.2	56.8	58.0	57.6	58.1
BRADA-TCA	65.1	66.9	61.5	60.8	59.7	58.9	62.2	61.7	68.3	69.1	63.1	65.7
BRADA-SA	63.5	63.8	60.9	59.6	58.7	59.9	60.0	59.5	64.6	65.2	63.0	65.6
BRADA-MIDA	65.6	68.1	59.9	63.2	60.6	59.8	61.7	63.1	69.9	69.6	63.5	69.2
BRADA	**71.0**	**74.9**	**68.9**	**66.7**	**65.4**	**69.8**	**65.8**	**69.1**	**75.5**	**78.3**	**71.9**	**73.8**

*The bold values indicated the highest accuracy obtained*.

**Table 5:** Cross-database classification accuracy (%) comparison with single brain region on MAHNOB-HCI and DEAP databases.

**Methods**	**Auditory channel**				**Visual channel**				**Auditory + Visual channels**			
	**A → B**		**B → A**		**A → B**		**B → A**		**A → B**		**B → A**	
	**Arousal**	**Valence**	**Arousal**	**Valence**	**Arousal**	**Valence**	**Arousal**	**Valence**	**Arousal**	**Valence**	**Arousal**	**Valence**
TCA (Pan et al., 2011)	53.7	51.1	58.6	53.2	47.1	50.7	53.3	54.2	54.1	51.7	60.2	55.8
SA (Fernando et al., 2013)	41.3	45.5	44.4	41.9	40.9	42.2	42.6	45.0	42.7	49.6	45.1	45.3
MIDA (Yan et al., 2018)	57.7	60.8	59.6	58.8	50.2	51.0	57.8	54.5	57.5	61.4	60.7	59.2
MIDA-POLY	56.6	58.9	50.3	54.4	46.6	45.2	50.2	49.8	56.8	59.1	51.4	55.1
BRADA-TCA	55.7	53.9	60.4	56.3	50.9	55.2	58.7	60.4	64.0	68.4	69.6	70.5
BRADA-SA	51.5	53.2	56.5	54.8	50.3	53.1	51.8	57.7	63.1	68.2	65.7	66.2
BRADA-MIDA	59.6	62.5	61.8	63.9	60.0	62.5	67.8	68.1	63.7	69.2	70.8	71.6
BRADA	**69.4**	**70.7**	**68.5**	**71.7**	**70.2**	**71.1**	**69.8**	**72.5**	**73.8**	**75.9**	**76.2**	**78.5**

*The bold values indicated the highest accuracy obtained*.

Another trend worth mentioning is that during the experiments, we observed that separating features into auditory and visual components independently and combining them together yields better performance than directly extracting features from all brain regions together. It also enables our algorithm in learning to adapt well and transfer knowledge effectively.

#### 3.3.2. Effect of Normalization

Our method handles features as random variables whose distribution is derived from previous subjects within a database. We introduce a procedure for normalization and augmentation of data which reduces variations within individual subjects. We show that normalization significantly improves the performance across subject-to-subject and database-to-database. The results are reported in Table 6. In our experiment, we observe that applying domain adaptation techniques directly to original features may result in a negative transfer.

**Table 6:** Classification accuracy (%) of BRADA with and without normalization.

**Methods**	**Cross-subject**				**Cross-database**			
	**MAHNOB-HCI**		**DEAP**		**A → B**		**B → A**	
	**Arousal**	**Valence**	**Arousal**	**Valence**	**Arousal**	**Valence**	**Arousal**	**Valence**
Without normalization	60.7	63.2	57.3	60.6	57.9	59.9	60.1	61.5
With normalization	**75.5**	**78.3**	**71.9**	**72.5**	**73.8**	**75.9**	**76.2**	**78.5**

*The bold values indicated the highest accuracy obtained*.

**Figure 4:**
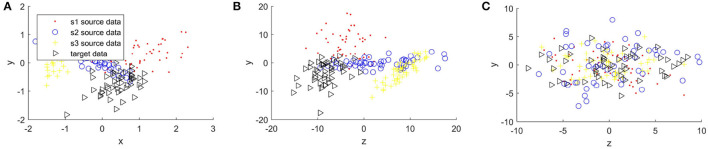
Sample distribution comparison of features in their original space and normalized projected spaces in the MAHNOB-HCI database, **(A)** is MAHNOB original features, **(B)** is Unnormalized features + brain region aware domain adaptation (BRADA), and **(C)** is Normalized features + BRADA.

**Figure 5:**
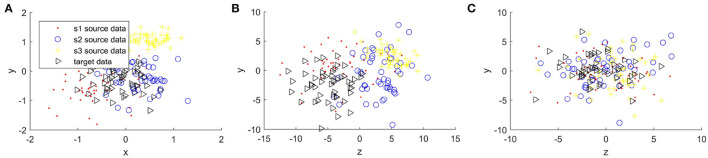
Sample distribution comparison of features in their original space and normalized projected spaces in the DEAP database, **(A)** is DEAP original features, **(B)** is Unnormalized features + BRADA, and **(C)** is Normalized features + BRADA.

Figures 4, 5 compare the sample distribution of unnormalized and normalized features projected in 2D space with BRADA. We also visualize the original data in 2D space by utilizing principal component analysis (PCA). Here, we used four subjects as an example. Three subjects being source data and one subject as target data. Figures 4A, 5A show the source and target domain features in their original space. As we can observe, feature samples are distributed differently between different participating subjects. Each subject as a source or target domain operates in its own space by belonging to separate clustered regions. This indicates a large divergence between individual subjects. Figures 4B, 5B shows sample distribution when data from the original space is directly applied to the BRADA algorithm. We observe that, in both databases, there was no significant effect in reducing discrepancies existing within subjects. Figures 4C, 5C show the feature sample distribution when features are normalized and augmented. Clearly, it can be seen that discrepancies and regional own space clusters are significantly reduced. Feature samples are better aligned on both databases suggesting that the domain adaptation algorithm was successful in somewhat learning structures across subjects and adapting to their spaces.

#### 3.3.3. Effect of Kernel Function Selection

**Figure 6:**
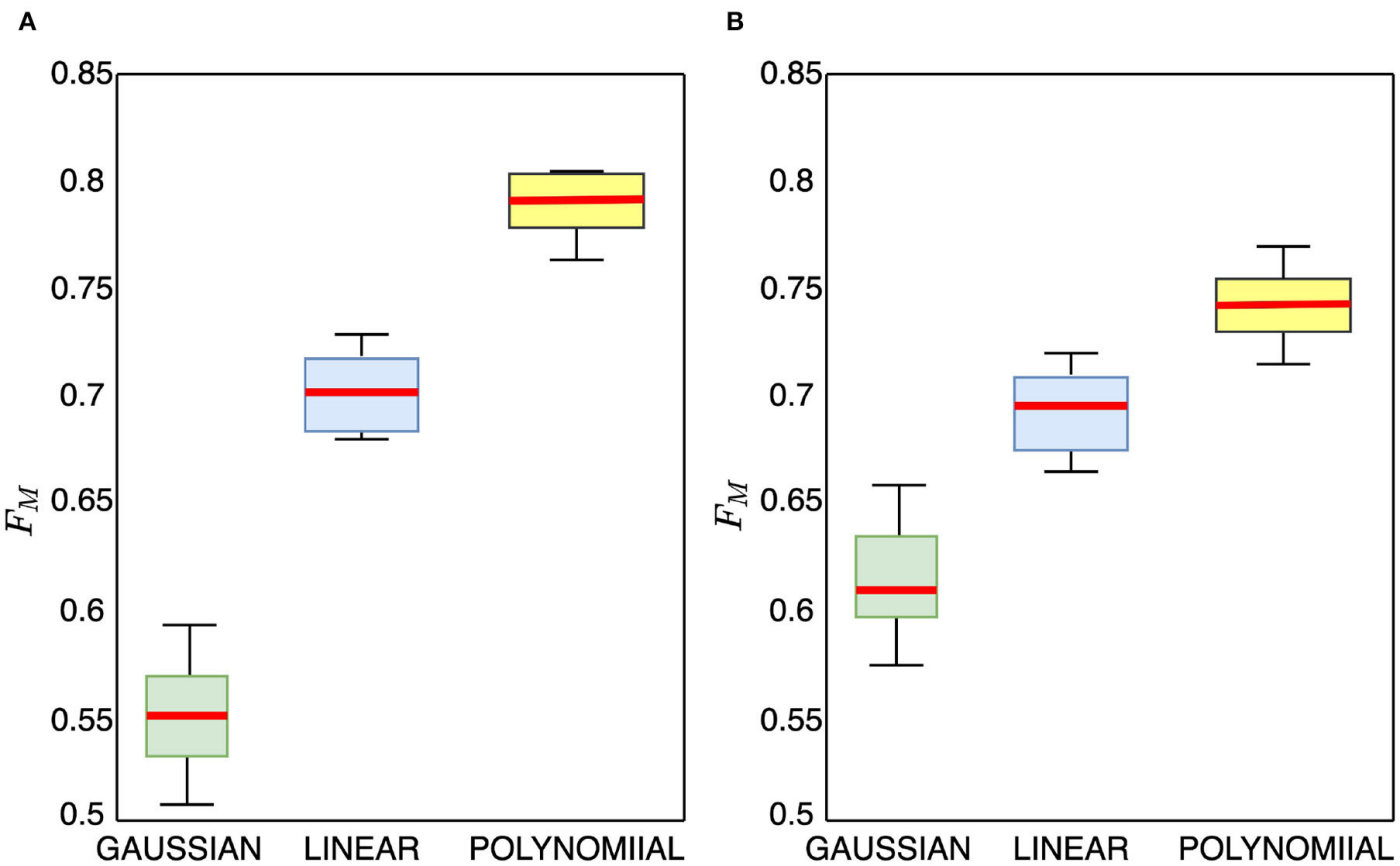
Box plots ablation experiment for valence cross-subject classification accuracy on MAHNOB-HCI and DEAP data using Gaussian, linear, and polynomial kernel mapping functions, **(A)** MAHNOB-HCI, **(B)** DEAP.

The compared TL methods utilize linear kernel function, while we employ polynomial kernel function. To further study the effectiveness of the polynomial kernel function, we compare it with the commonly used Gaussian and linear kernel functions and show the box plots in Figure 6. From the figure, we observe that the Gaussian kernel function approach achieves the lowest performance results. The polynomial kernel function achieves significantly statistically better performance than the linear kernel function with no overlaps seen. The polynomial kernel obtains a higher median accuracy and also produces a narrower box than the linear kernel indicating that the polynomial kernel reduces variations beneficial for transfer learning. Worth noting, we observe during the experiment that applying a polynomial kernel improves both recall and precision of the model's performance on the test data in both cross-subject and cross-database classification proving that our method returns relevant results.

## 4. Discussion

**Figure 7:**
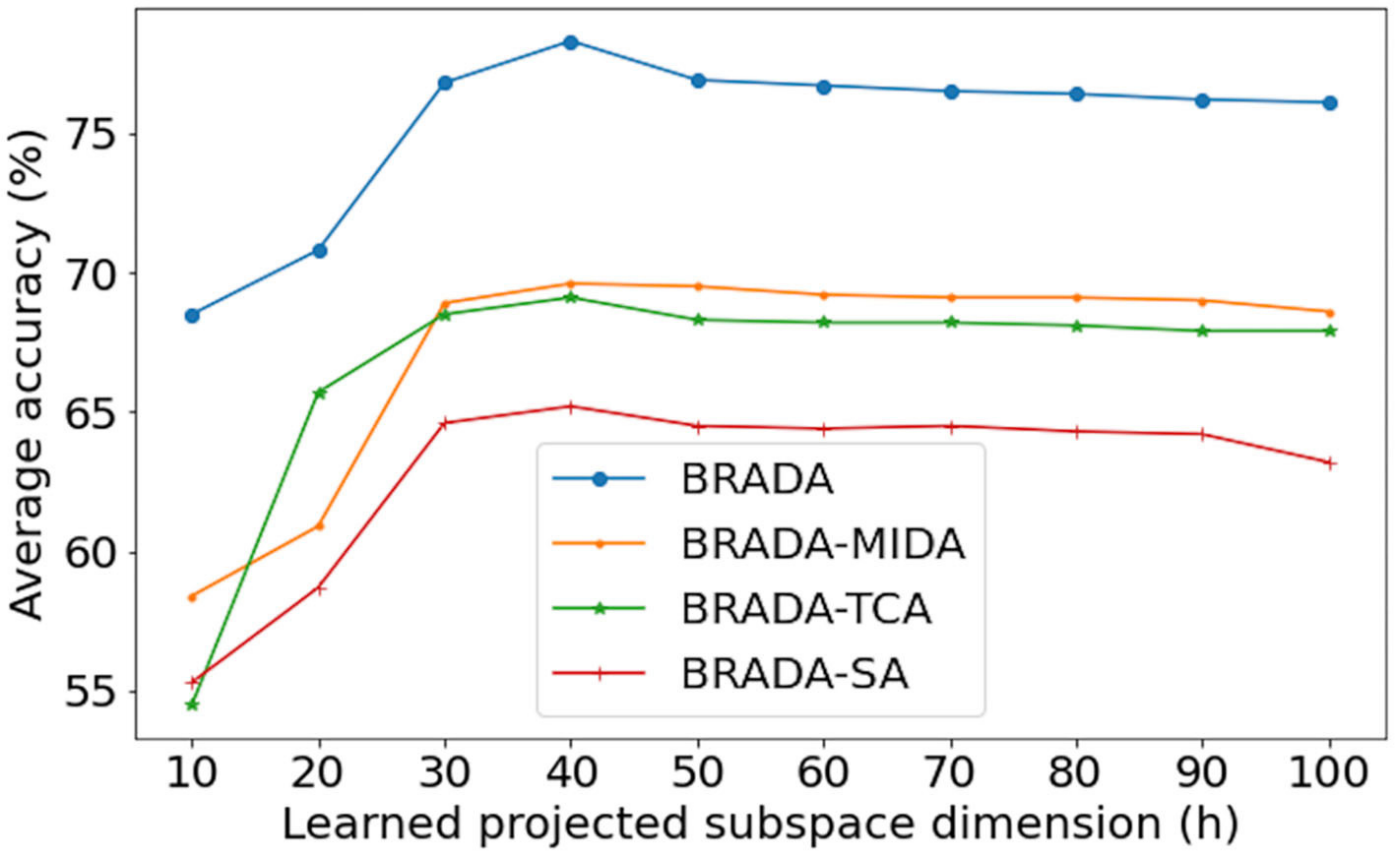
Average valence performance accuracy on MAHNOB-HCI using varying projected dimensions.

The dimension number *h* of the latent space is a critical hyper-parameter of domain adaptation methods. It is hard to determine the optimal dimension directly because the intrinsic dimension is affected by channel numbers and the kind of feature. To study the effect of feature dimension, we show the average valence classification accuracy of all BRADA variants on MAHNOB-HCI under cross-subject settings with varying learned projected subspace dimension *h* in Figure 7. The classification accuracy increases as the dimension increases at the lower dimension and becomes stable when the dimension is large enough. We can see that all BRADA variants achieve the best performance when *h* = 40. This implies that the feature has an intrinsic dimension of around 40. The cross database strategy identifies that the conventional aBCI prototype cannot be fully satisfied. The BRADA algorithm effectively copes with domain and technical discrepancies. This is of practical sense as it can reduce constraints on aBCI and develop into clinical applications and novel therapies for stress, depression, and other nervous system disorders. Therefore, more future studies are needed on this topic.

This article argues not all electrode channels are needed and investigated the effects of auditory and visual channels on two EEG databases for emotion recognition. We propose a multi-source and multi-target transfer learning method that first applies domain adaptation to different brain regions separately and then combines them. The method reduces inter-subject discrepancy and demand for data calibration effectively. It also can be used to train one database and test on a distinct but related database. Experimental results show the superiority of our approach.

## Data Availability Statement

Publicly available datasets were analyzed in this study. This data can be found here: https://mahnob-db.eu/hci-tagging/ and https://www.eecs.qmul.ac.uk/mmv/datasets/deap/.

## Ethics Statement

Ethical review and approval was not required for the study on human participants in accordance with the local legislation and institutional requirements. The participants provided their written informed consent to participate in this study.

## Author Contributions

HP, XXi, KG, and XXu proposed the idea. HP conducted the experiment, analyzed the results, and wrote the manuscript. XXi was in charge of technical supervision and provided revision suggestions. KG analyzed the results, reviewed the article, and was in charge of technical supervision. XXu was in charge of technical supervision and funding. All the authors contributed to the article and approved the submitted version.

## Funding

This work was supported in part by the National Natural Science Foundation of China under Grant U1801262, Guangdong Provincial Key Laboratory of Human Digital Twin (2022B1212010004), in part by Science and Technology Program of Guangzhou under Grant 2018-1002-SF-0561, in part by the Key-Area Research and Development Program of Guangdong Province, China, under Grant 2019B010154003, in part by Natural Science Foundation of Guangdong Province, China, under Grants 2020A1515010781 and 2019B010154003, in part by the Guangzhou key Laboratory of Body Data Science, under Grant 201605030011, in part by Science and Technology Project of Zhongshan, under Grant 2019AG024, and in part by the Fundamental Research Funds for Central Universities, SCUT, under Grants 2019PY21 and 2019MS028.

## Conflict of Interest

The authors declare that the research was conducted in the absence of any commercial or financial relationships that could be construed as a potential conflict of interest.

## Publisher's Note

All claims expressed in this article are solely those of the authors and do not necessarily represent those of their affiliated organizations, or those of the publisher, the editors and the reviewers. Any product that may be evaluated in this article, or claim that may be made by its manufacturer, is not guaranteed or endorsed by the publisher.
